# Structural Propensities of Human Ubiquitination Sites: Accessibility, Centrality and Local Conformation

**DOI:** 10.1371/journal.pone.0083167

**Published:** 2013-12-11

**Authors:** Yuan Zhou, Sixue Liu, Jiangning Song, Ziding Zhang

**Affiliations:** 1 State Key Laboratory of Agrobiotechnology, College of Biological Sciences, China Agricultural University, Beijing, China; 2 National Engineering Laboratory for Industrial Enzymes and Key Laboratory of Systems Microbial Biotechnology, Tianjin Institute of Industrial Biotechnology, Chinese Academy of Sciences, Tianjin, China; 3 Department of Biochemistry and Molecular Biology, Faculty of Medicine, Monash University, Melbourne, Victoria, Australia; University of Alberta, Canada

## Abstract

The existence and function of most proteins in the human proteome are regulated by the ubiquitination process. To date, tens of thousands human ubiquitination sites have been identified from high-throughput proteomic studies. However, the mechanism of ubiquitination site selection remains elusive because of the complicated sequence pattern flanking the ubiquitination sites. In this study, we perform a systematic analysis of 1,330 ubiquitination sites in 505 protein structures and quantify the significantly high accessibility and unexpectedly high centrality of human ubiquitination sites. Further analysis suggests that the higher centrality of ubiquitination sites is associated with the multi-functionality of ubiquitination sites, among which protein-protein interaction sites are common targets of ubiquitination. Moreover, we demonstrate that ubiquitination sites are flanked by residues with non-random local conformation. Finally, we provide quantitative and unambiguous evidence that most of the structural propensities contain specific information about ubiquitination site selection that is not represented by the sequence pattern. Therefore, the hypothesis about the structural level of the ubiquitination site selection mechanism has been substantially approved.

## Introduction

The fate of many eukaryotic proteins is controlled by the ubiquitination process [1,2], in which a targeted protein is conjugated with small protein ubiquitins that are organized as either monomers or polymer chains of certain topology [3]. The information embedded in the conjugated ubiquitins is generally deciphered by the ubiquitin binding domains [4], as such the degradation, localization or interaction of the targeted protein is regulated accordingly [5]. Human protein ubiquitination has also been reported to be associated with a number of diseases like Huntington's disease [6], breast cancer [7] and acquired immune deficiency [8].

Despite early awareness of a wide range of biological processes regulated by ubiquitination [9], only with recent breakthrough in proteomic techniques can the widespread ubiquitination sites (Ubsites) in the human proteome be extensively characterized in the large-scale studies [10-14]. These experiments have revealed unique features of Ubsites in comparison with other post-translational modification (PTM) sites. On the one hand, in addition to the topology of ubiquitin chains, the selection of which lysines in the substrate protein to be ubiquitinated is non-trivial. The amino acid pattern in the context (i.e. the flanking sequences) of human Ubsites appears to be discernible [10,12,13] and has been exploited to predict human Ubsites with acceptable accuracy [15-17]. On the other hand, in contrast to the primary hypothesis of ubiquitination motifs [18,19], which are in analogy to phosphorylation motifs that determine phosphorylation site specificity, human Ubsites exhibit noticeable variability during evolution and characteristic ubiquitination motifs are hard to find [10,13]. Altogether, these results have motivated us to investigate the preferences of human Ubsites from an alternative and potentially insightful, structural perspective.

Large-scale computational structural analyses can provide valuable insights into the underlying mechanism and functional impacts of PTMs. Such analyses have become feasible with the rapid growth of protein 3D structural data. For example, an extensive analysis of phosphorylation sites revealed distinguishable amino acid preferences in their structural neighbors [20]. Based on the calculations of binding energy change, the stronger influence of phosphorylation on the formation and stability of transient protein complexes was closely investigated and quantified [21]. Through the comparison of multiple structures of the modified proteins, the significant influences of PTMs on the protein conformation dynamics were discovered [22]. Despite the aforementioned success of computational structural analyses of other types of PTMs, little knowledge about Ubsites has been gained from the substrate structure. To the best of our knowledge, Catic et al. carried out the only pioneering study to investigate yeast Ubsites in protein structures. They observed higher solvent accessibility and preference for random-coil of yeast Ubsites using a small set of 23 protein structures [18]. However, further quantification and extensive validation of these observations were prohibited by the limited amounts of data at that time. Instead, we are encouraged by a recent study showing that human Ubsites, unlike their yeast counterparts [23], can be frequently mapped to structured domains [24].

In this study, we have performed a systematic analysis of 1,330 human Ubsites in 505 PDB chains. Our analysis confirms and further quantifies the higher accessibility of human Ubsites with various parameters like the relative accessible surface area (RSA) and the protrusion index. Besides, our results suggest that the centrality emerges as a novel trait of Ubsites and we have extensively analyzed and discussed its implication for the wide functional associations of Ubsites. Third, we compare the information included in the sequence context and the structural microenvironment in detail. Finally, we demonstrate the complementary relationship between the sequence pattern and the structural propensities in discriminating Ubsites from non-ubiquitination sites (Non-Ubsites).

## Materials and Methods

### Dataset

The human Ubsites identified from five recent proteomic assays [10-14] were mapped onto the UniProt [25] protein sequences (release 2012_09). To achieve high confidence, only Ubsites identified by at least two experiments were retained. Moreover, this dataset was further enriched by including the human Ubsites manually curated from literature by UniProt [25], Hagai et al. [26] and our group [16]. Lysine residues that have not been annotated by any of the aforementioned five proteomic assays or through literature search were initially treated as Non-Ubsites. The Non-Ubsite data were further filtered against the Ubsites collected by the PhosphoSitePlus^®^ database [27] (http://www.phosphosite.org).

The Ubsites and Non-Ubsites were further mapped onto the structures in PDB (http://www.pdb.org) to obtain their structural information. The redundant (sequence identity>50%), mutant or low resolution (worse than 4.0 Å or missing all side-chain atom coordinates) PDB chains were discarded. We also restricted the retained PDB chains to have at least one Ubsite and one Non-Ubsite. Thus, PDB chains that contain Ubsites only (e.g., the ubiquitin itself) were also abandoned. As a result, 1,330 Ubsites and 5,465 Non-Ubsites were mapped onto the 505 PDB structures (Table S1), which cover 151 folds and 229 families according to the latest SCOP [28] annotations. To facilitate the analyses, we further established the numbering correspondence between the residues in the PDB chains and those in the Uniprot sequences, and removed the unmapped residues (e.g. protein expression tags and alternatively spliced regions). In the case of alternative conformations of the same residue or multiple structure models of the same chain (i.e. the case of 68 chains solved by NMR), only the first one was kept. We noted that 17 Ubsites and 53 Non-Ubsites in the NMR structures exhibit large conformation flexibility (i.e. average C_α_ RMSD>5.0 Å). It is possible that these residues are in disordered state and may not be included in the structural analysis. However, because these residues comprise only a small fraction (about 1%) of our dataset, our conclusions are unlikely to change if these high flexible residues are removed. The hydrogen atoms were removed to avoid the confusion of some analytical programs used in this study. We also noted that some modified residues that were presented as HETATM records in the PDB files could be ignored by some analytical programs. Thus, we restored these modified residues to their unmodified ATOM records following the guidance of PDB annotations.

### Statistical Tests

Unless stated otherwise, Wilcoxon test and Fisher’s exact test were used for two sample value comparison and enrichment test, respectively. We also report the effect size *r* for Wilcoxon test to estimate the amplitude of the difference between two samples. An *r* value around -0.1 indicates a small but observable difference. All statistical tests were performed in R (http://www.r-project.org).

### Accessibility Calculation and Residue Contact Network Analysis

The RSA was calculated by the NACCESS software (http://www.bioinf.manchester.ac.uk/naccess/) with an upper-bound value of 100. We further introduced 918 acetylation sites collected from the PhosphoSitePlus^®^ database [27] as the positive control in the RSA analysis 2 alternative accessibility parameters, i.e. the protrusion index CX and the depth index DPX [29], were calculated using PSAIA [30]. By calculation, each atom in a residue was assigned with one pair of CX and DPX. We chose the maximum CX value and the average DPX value for a residue to depict its protrusion and depth due to higher discriminative power (alternative choices do not affect the conclusion; see Figure S1A and B).

In a Residue Contact Network (RCN), one pair of contacting residues are depicted as two nodes connected by one edge. The RCN was constructed by defining two residues as a contacting residue pair if the distance between their C_β_ atoms (C_α_ for glycine) was less than 7.5 Å [31]. We also validated the results using an alternative definition of the residue contact, where two residues were considered as a contacting pair if the distance between any two atoms from each residue was smaller than 4.0 Å [32]. Two key network topology parameters, degree and closeness centrality, were extracted from the networks using the igraph package [33] in R. In terms of topology interpretation, the degree of a node measures how many nodes are connected to it, while the closeness centrality depicts how few steps are required to move from one node to all other nodes throughout the network [33]. The physicochemical interpretation of these two parameters is more straightforward: high degree residues are densely packed [34], and residues with high closeness centrality are located near the geometric center of a protein [35]. To validate the closeness centrality, we also calculate the distance for each Ubsite/Non-Ubsite to the protein geometric center. One may refer to the Text S1 for the detailed calculation.

### Functional Site Annotations

The catalytic sites were assigned by the Catalytic Site Atlas database [36]. The POCKET software [37] was utilized to perform ligand binding pocket prediction, and only the largest pocket in each structure was considered. We used a computational alanine scan method provided by the FoldX software [38] to measure the contribution of a lysine residue to protein folding (see also Text S1). Ideally, a folding hotspot residue can be identified if its mutation to alanine results in a significant energetic loss of the folded protein (ΔΔG>2 kcal/mol).

The protein complex structures were constructed according to the REMARK350 records in the PDB file (which describe how monomer structure should be duplicated, moved and rotated to establish the complex structure). The 3D-complex database [39] was employed as a supervisor of the construction process. 290 protein complex structures carrying at least one ubiquitination site were constructed. A residue was considered as interface residue if the difference of its solvent accessible surface area between the monomer state and the complex state (i.e. ΔASA) was larger than 5 Å^2^. We further grouped the protein complexes according to their stability [40] and calculated the propensity of Ubsites being located on the interfaces for each group (see Text S1).

### Secondary Structure, Structural Alphabets and Microenvironment

The eight-type secondary structure and 22-state structural alphabet [41] were calculated by DSSP [42] and our in-house program, respectively. The structural alphabet is a classification of protein local conformation state based on the κ and α angles formed by the neighboring C_α_ atoms [41]. Note that structural alphabet states “Y” and “A” were merged as suggested in the original work [41]. See Table 1 and Table 2 for the lists of secondary structure types and structural alphabet states, respectively. Using the TwoSampleLogo tool [43], we plotted the logo illustrations that indicate the enriched and depleted residues, secondary structure types or structural alphabet states at each position in the context (i.e. the sequence neighbors).

**Table 1:** The secondary structure types.

Type	Description
H	α-helix
G	3^10^-helix
I	π-helix
E	β-bulge
B	β-bridge
T	turn
S	highly curved coil
L	loop (other coils)

**Table 2:** The structural alphabet states.

State	Description
A	helix conformation
B	helix conformation
C	helix conformation
D	helix conformation
G	helix-like conformation
I	helix-like conformation
L	helix-like conformation
E	strand conformation
F	strand conformation
H	strand conformation
K	strand-like conformation
N	strand-like conformation
M **^*a*^**	highly curved coil **^*b*^**
S	highly curved coil **^*b*^**
V	highly curved coil **^*b*^**
W **^*a*^**	highly curved coil **^*b*^**
Q	moderately curved coil **^*b*^**
R	moderately curved coil **^*b*^**
T	flat coil **^*b*^**
P	flat coil **^*b*^**
X	flat coil **^*b*^**
Z **^*a*^**	flat coil **^*b*^**

^a^ These structural alphabet states showed no over- or under-representation in the context of the ubiquitination sites in our dataset.

^b^ The coil conformations were classified into three groups: highly curved coil, moderately curved coil and flat coil. Note that this classification was not proposed by the original research, but by us in this study according to the similarity of the local conformations they represent.

In addition to the context, the microenvironment (i.e. the structural neighbors) of a functional site may also exhibit distinguishable residue usage. One example is the case of enzyme catalytic sites [35]. In this study, we defined a three-shell microenvironment according to the C_β_ distance from a central lysine to its neighboring residues: 0~7.5Å for the first shell, 7.5Å~11.5 Å for the second shell and 11.5 Å~15.5 Å for the third shell. The residue propensity in each shell is calculated as the residue’s frequency in this shell divided by its frequency in the whole structure.

### Analyzing the Ubiquitination Site Indicators via ROC Curve

We used the closeness centrality value and the CX value as the centrality indicator and accessibility indicator, respectively. The CX values were linearly scaled into the range of 0~1 for the comparison [35]. For other indicators like sequence pattern, local conformation frequencies or residue propensities in the microenvironment, the likelihood scores were derived from either Naïve Bayes model or random forest model via five-fold cross-validation (see Text S1).

The receiver-operating characteristic (ROC) curves were plotted based on the indicators (propensity values and likelihood scores). We also plotted the ROC curves for the combination of indicators based on the combined scores. The combined scores are the sum of the parameter values and the likelihood scores with preliminary optimized weightings (Table S2). The area under the ROC curve (AUC) was also calculated for individual indicators and the combined scores, in order to measure their capabilities to discriminate Ubsites from Non-Ubsites. Intuitively, the higher the discriminative capability of one indicator is, the larger AUC can be measured. If two indictors strongly complemented each other, a significant augment of AUC would be observed when they were combined. The statistical significance of the difference between two AUC values was tested by DeLong’s test from the pROC package [44] in R.

## Results and Discussion

### Higher accessibility and centrality of human ubiquitination sites

**Figure 1:**
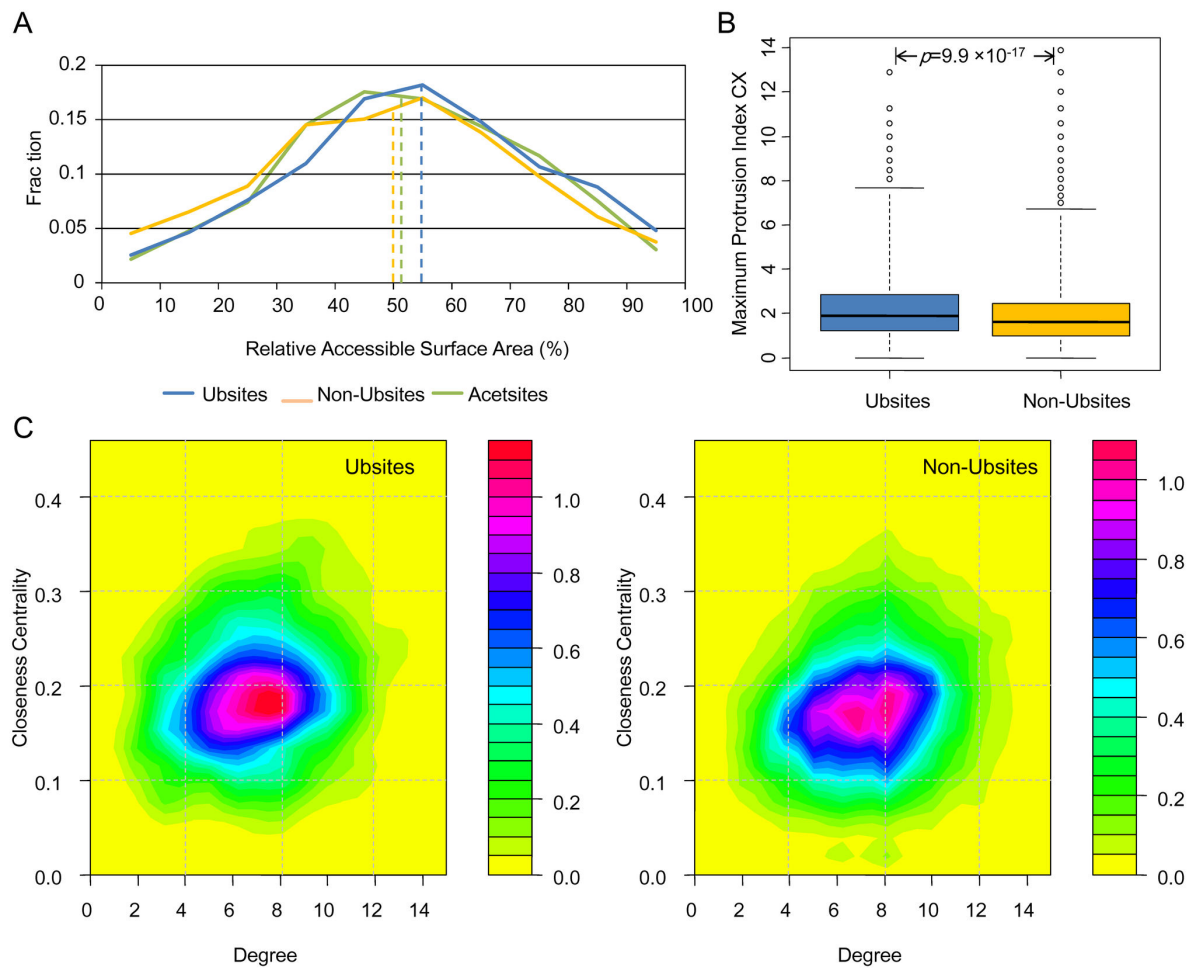
The accessibility and centrality of the ubiquitination sites. (A) Distribution of RSA for Ubsites, Non-Ubsites and Acetsites. The median values are indicated as vertical dashed lines. (B) Boxplot depicting the difference in the maximum protrusion index CX between Ubsites and Non-Ubsites. The range of whisker (dashed lines) is doubled to avoid displaying too many outliers. (C) Two-dimensional probability density plots illustrating the propensity for two network parameters of Ubsites (left) and Non-Ubsites (right). Note that the range and color schemes of these two plots have been unified in order to make a direct comparison.

We started with the RSA analysis of Ubsites and Non-Ubsites. As can be seen in Figure 1A, the vast majority (92.8%) of Ubsites tend to be exposed to the solvent (with an RSA>20%). Statistical test confirmed a distribution shift toward higher RSA for Ubsites compared with Non-Ubsites (*p*=2.9×10^-10^). However, while some other PTM sites like phosphorylation sites exhibit highly prominent discrepancy in accessibility compared with non-modified residues [45], the discrepancy between Ubsites and Non-Ubsites seems not obvious at first glance (effect size *r*=-0.075). In contrast to phosphorylation substrate residues (S, T and Y), lysines are unlikely to be buried due to their charged nature. Thus, one may fail to observe prominent discrepancy in RSA between Ubsites and Non-Ubsites, as the RSA of Non-Ubsites should also be high. In fact, Ubsites show a slightly higher RSA even compared with the acetylation sites [Another type of important lysine PTM [46]; Acesites in Figure 1A, *p*=0.027], which further confirms the high accessibility of Ubsites.

It has been previously observed, in a small set of 23 structures, that yeast Ubsites tend to be highly accessible [18]. Our results quantitatively consolidated this observation. Moreover, we found the protrusion index CX and the depth index DPX could also discriminate Ubsites from Non-Ubsites. Ubsites tend to have remarkably higher CX (Figure 1B, *p*=9.9×10^-17^, *r*=-0.10) and lower DPX (Figure S1C, *p*=3.1×10^-5^, *r*=-0.049). These results imply that Ubsites are highly protruding and less buried, making them readily accessible to solvent and ubiquitination enzymes.

We further analyzed the location of Ubsites utilizing the degree and closeness centrality parameters from RCNs. Our results indicated that Ubsites have lower degree (*p*=2.7×10^-7^, *r*=-0.061) compared with Non-Ubsites, which is in agreement with their lower DPX. Unexpectedly, however, Ubsites show significantly higher closeness centrality compared with Non-Ubsites (*p*=3.0×10^-18^, *r*=-0.10). This is an exceptional observation because the closeness centrality shows a positive correlation with the degree parameter in our dataset (correlation coefficient=0.18, *p*<10^-50^). The differences in degree and closeness centrality are also clearly reflected by the two-dimensional probability density maps (Figure 1C). A considerable fraction of Non-Ubsites are localized in the region of degree larger than 8, but this region is less favored by Ubsites. The discrepancy is more significant for closeness centrality: Ubsites are aggregated in the region with closeness centrality of about 0.18, resulting in a holistic upper-shifted distribution compared with Non-Ubsites. The higher closeness centrality was confirmed with an alternative definition of residue contact (Figure S1D). The closeness centrality can also be explained as the geometric centrality, that is, Ubsites prefer to be located closer to the geometric centers of proteins (*p*=1.5×10^-9^, *r*=-0.072; Figure S2A). One may note that the absolute distance between a Ubsite/Non-Ubsite and the protein geometric center should be partly correlated with protein size. Nevertheless, after corrected for the protein size, Ubsites still showed closer localization to the geometric centers of proteins (*p*=3.9×10^-7^, *r*=-0.060; Figure S2B), confirming the higher centrality of Ubsites. As many protein functional sites also tend to locate at the geometric centers of proteins, the centrality has been further shown to be indicative of a wide spectrum of protein functional sites [35,47,48]. Therefore, it is of particular interest to test if Ubsites are associated with certain functional sites in the structures. We investigated the relationship between Ubsites and multiple functional sites, which is detailed in the next section.

### Potential Functional Impacts of Ubiquitination Sites

#### Ubiquitination Sites and Enzyme Catalytic Sites

We first examine the relationship between the enzyme catalytic sites and Ubsites, because the enzyme catalytic sites showed the strongest association with centrality among several types of functional sites [47]. We used both experimental and predicted catalytic sites from the Catalytic Site Atlas database [36] since the experimental ones are not always available. In this way, we assigned catalytic sites for 88 PDB chains (enzymes) in our dataset. Indeed, Ubsites are generally located closer to the catalytic residues (C_β_ distance, *p*=0.0041, *r*=-0.044). Nevertheless, the absolute distance between a Ubsite and a catalytic site should be close enough to let the attached ubiquitin molecules block the catalytic site directly. Accordingly, we set a C_β_ distance cutoff of 11.5 Å (which is approximately the radius of ubiquitin) to define direct association. By this definition, only 31 Ubsites are directly associated with the catalytic residues, and show no relative enrichment (Fisher’s exact test, *p*>0.2). Similar results could be obtained if a more stringent cutoff of 7.5 Å was adopted (data not shown). Therefore, we conclude that direct association with the enzyme catalytic site is not likely the exclusive way for Ubsites to influence enzyme activities. Instead, some Ubsites may regulate the enzyme activity in indirect fashions. We will test this hypothesis in the next sub-section.

#### Ubiquitination Sites and Ligand Binding Sites

**Figure 2:**
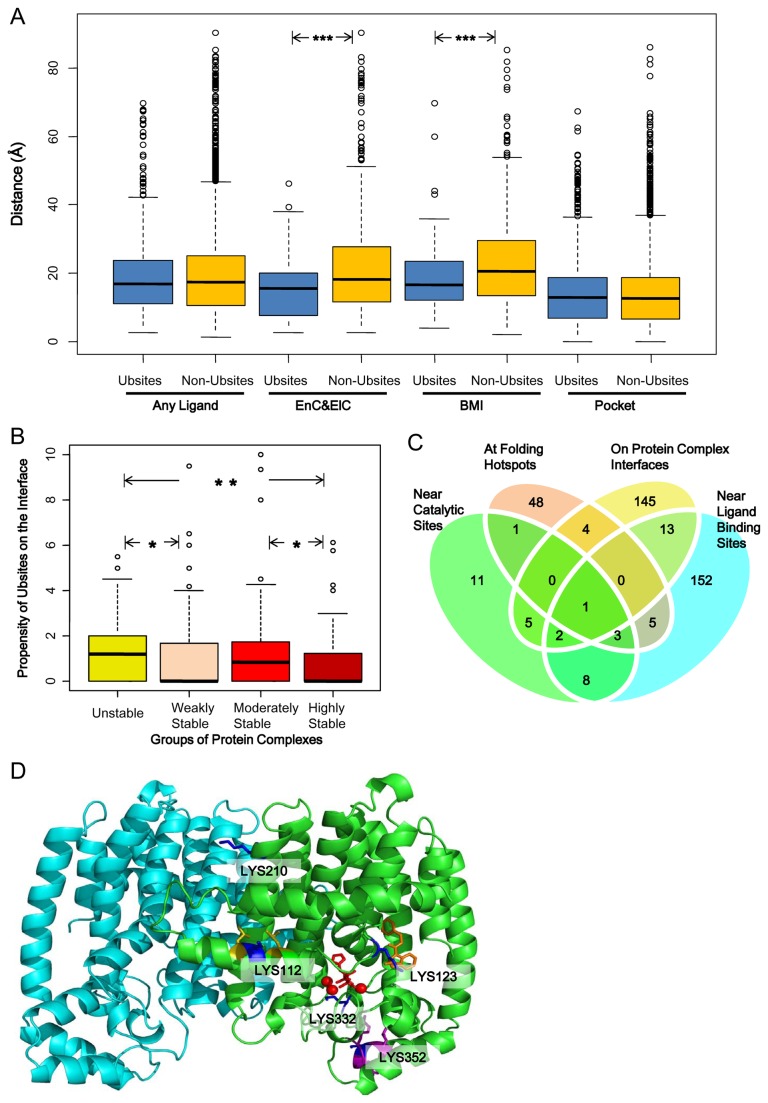
Wide association between ubiquitination sites and functional sites in the structures. (A) Boxplot showing the distribution of the shortest distance between Ubsites/Non-Ubsites and the ligands/largest pockets. EnC&ElC, energy currency and electron carrier; BMI, bivalent metal ion. (B) Boxplot showing difference in the propensity of Ubsites on the interface between different groups of complexes. The statistical significance in panel A and B (if any) is labeled as: *, *p*<0.05; **, *p*<0.01, ***, *p*<0.001. (C) The Venn diagram shows the overlap of the Ubsites that are associated with different types of functional sites. (D) The cartoon representation of the farnesyl pyrophosphate synthase, on which five Ubsites (blue) are highlighted. Red stick, the inhibitor zoledronic acid targeting the substrate pocket; red ball, Mg^2+^ ions; orange stick, an allosteric inhibitor; yellow residues, two folding hotspots; purple residues, the KEN motif.

In our dataset, 236 out of 505 PDB chains bind at least one ligand. However, the shortest distances between Ubsites and the ligands are not significantly smaller compared with Non-Ubsites (*p*>0.2). This result would be an underestimation considering that the ligands were not always presented in the structures. To better understand this, we predict the presence and location of ligand binding site (i.e. the largest pocket) on the structure. However, no clue for closer distance between Ubsites and ligand binding pockets was found (*p*>0.2). Therefore, Ubsites are more likely to be associated with specific types of ligands only. Through a careful investigation, we found that Ubsites were located significantly closer to two types of ligands (Figure 2A), namely energy currency & electron carriers (e.g., ATP and NADP; *p*=5.2×10^-4^, *r*=-0.15) and bivalent metal ions (e.g., Zn^2+^; *p*=3.1×10^-4^, *r*=-0.14). We have shown above that direct association between Ubsites and the catalytic sites is not widespread. By contrast, 52 Ubsites appear to be directly associated with these specific ligands (shortest distance<11.5 Å), accounting for 28% of all ligand-associated Ubsites. As these ligands often play a role as enzyme co-factors *in vivo* [49], it is plausible that for some enzymes ubiquitination regulates their activity via the regulation of co-factor binding, instead of the direct blockage of the catalytic sites.

#### Ubiquitination Sites and Folding Hotspots

Protein unfolding may be a prerequisite for ubiquitination-mediated protein degradation, because the catalyzing enzyme complex 26S proteasome has a narrow substrate translocation channel [50]. As a consequence, one tends to speculate that the conjugated ubiquitins themselves can induce protein unfolding to help the attached substrates pass through this narrow channel. Computational molecular simulation of a yeast protein supported this idea that the protein folding could be substantially disrupted when being conjugated with ubiquitin chains [51]. But whether ubiquitination tends to target residues important for folding stability (i.e. the folding hotspots) has not been tested. According to the results of computational alanine scan, no larger energy contribution of Ubsites was indicated, as Ubsites have lower energy contribution on average (ΔΔG, 0.55 kcal/mol *vs.* 0.60 kcal/mol, *p*=0.0042). Furthermore, Ubsites do not seem to favor folding hotspots: only 3.0% of Ubsites correspond to the folding hotspots, while the fraction is slightly higher (3.6%) for Non-Ubsites. Nevertheless, it should be noted that in principle our results neither approved nor declined the role of ubiquitin as a destabilizer of protein folding. Instead, the results highlight potentially extensive functional impacts of ubiquitination where the folding hotspots targeted by ubiquitination represents only a small portion of the functional sites that may be influenced by ubiquitination.

#### Ubiquitination Sites and Protein-protein Interaction Sites

Generally, 170 out of 884 Ubsites in the protein complexes settle on the interface, but this fraction is only marginally higher compared with Non-Ubsites (*p*=0.039). This indicates that only few subsets of complexes are relatively enriched for Ubsites on their interfaces. Similar to [21], we grouped the complexes to four different groups (unstable, weakly stable, moderately stable and highly stable) based on their stability and found that the interfaces of unstable complexes seem to be the most favorable target for ubiquitination (Figure 2B). However, this result is not statistically significant probably because of the small sample size available. The unstable complexes are usually maintained by transient protein-protein interactions, which are also likely to be regulated by other PTMs like phosphorylation [21]. Therefore, it is interesting to ascertain if Ubsites tend to be located on the interface core (ΔASA>85 Å^2^) to unleash a strong regulatory capability. We found that Ubsites are generally located on the rim of the interfaces (ΔASA<25 Å^2^), even for the unstable complexes (Figure S3A). However, a noticeable subset of Ubsites instead favor the interface core of the unstable complex (Figure S3A, yellow line). This phenomenon was not observed for Non-Ubsites (Figure S3B), indicating that the Ubsites play at least a partial role in regulating the transient association of unstable complexes. By contrast, the interface cores of highly stable complexes seem to avoid being ubiquitinated (Figure S3A). This tendency can be attributed to the difficulty of these highly stable complexes to be dissociated to expose a ubiquitination substrate lysine on their interface core.

#### Multi-functionality of Ubiquitination Site

Taken together, the association between Ubsites and specific functional sites has been observed. Our results also complement the computational analyses of Ubsite function that were rooted from the evolutionary conservation [26]. However, as shown in Figure 2C, Ubsites seemed to influence various types of functional sites, which rarely overlap with each other in most cases. These results suggest that the broad spectrum of functional sites that can be influenced by Ubsites.

An example for the multi-functionality of Ubsites is showcased by the farnesyl pyrophosphate synthase (PDB entry: 3N45). This dimeric enzyme can catalyze sequential reactions to produce farnesyl pyrophosphate [52]. The inhibition of this enzyme is of clinical significance as its product can serve as not only intermediates for several metabolic pathways, but also substrates for a few PTMs like farnesylation [52,53]. Five Ubsites (LYS332, LYS123, LYS112, LYS210 and LYS352) scattered on the enzyme’s structure, and each has a distinct potential functional impact, either direct or indirect (Figure 2D). LYS332 is located at the bottom of the enzyme substrate pocket, with a close distance (5.5 Å) to the cofactor Mg^2+^ ions. LYS123 does not point to the substrate pocket, but stretches into the allosteric pocket and binds the allosteric inhibitor [53]. LYS112 lies in a densely packed region accompanied by two folding hotspots. Though it has only moderate folding energy contribution itself, it may play a role in the communication of the two neighboring hotspots. LYS210 is on the dimer interface, but it is excluded from the interface core like many other Ubsites in stable complexes. Finally, LYS352 is located away from the aforementioned typical functional sites in this structure. Instead, it appears to be a key component of the KEN motif that mediates protein degradation [54].

### The Context and Microenvironment of Ubiquitination Sites

**Figure 3:**
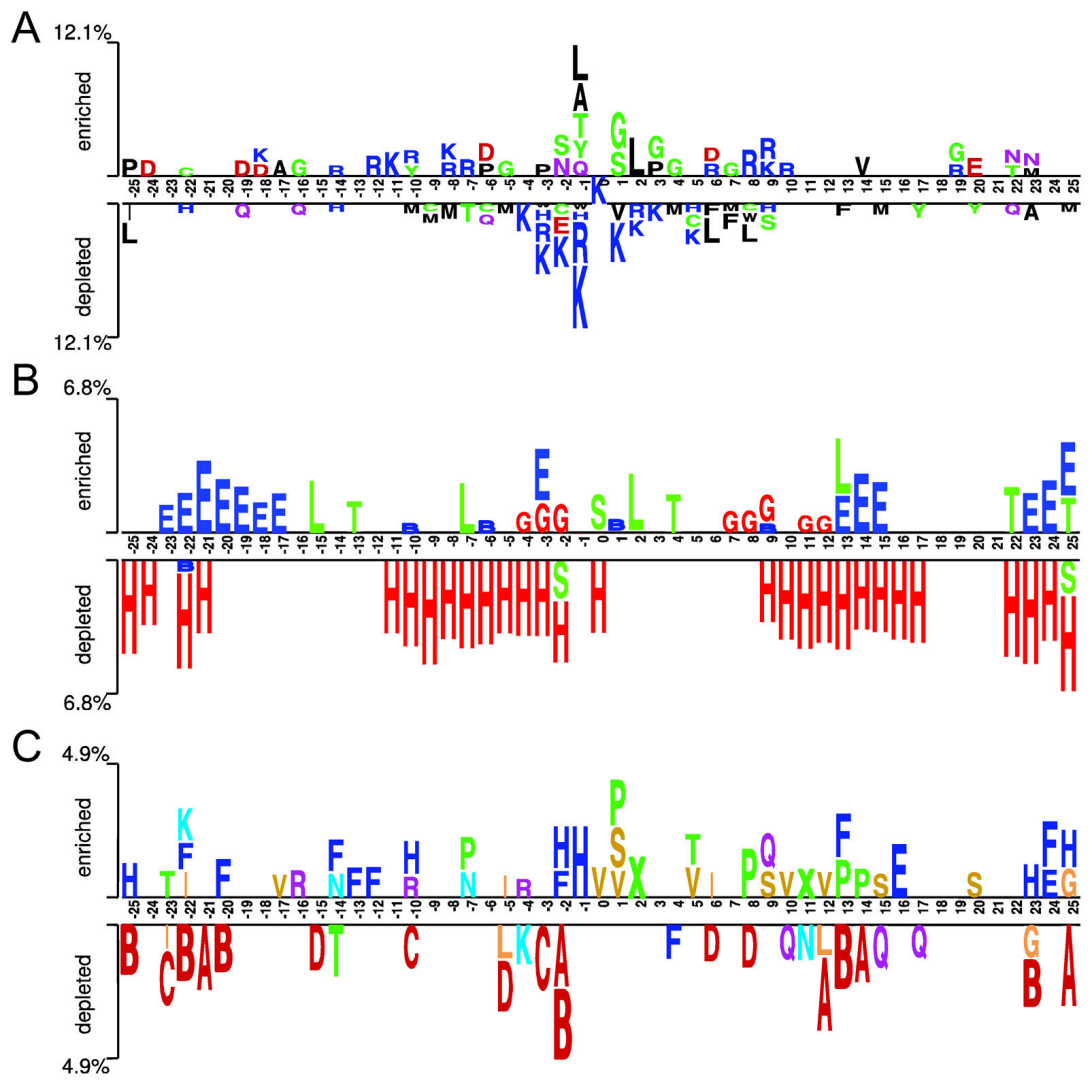
The two-sample logo illustration of the context (sequence neighbors) of ubiquitination sites. (A) The positional residue pattern; (B) the secondary structure pattern and (C) the local conformation (structural alphabet) pattern where a seven-group color palette is used: helix (red), helix-like (orange), strand (blue), highly curved coil (yellow), moderately curved coil (violet) and flat coil (green). See also Tables 1 and 2 for the description of the secondary structure type and structural alphabet state, respectively.

The context (sequence neighbors) and/or the microenvironment (structural neighbors) of a functional site often have specific sequence and structural preferences. It has been widely accepted that the sequence pattern in the context is the most distinguishable signature of Ubsites [12,13,15-17,23]. As shown in Figure 3A, the sequence logo representations of ±25 residues around Ubsites. As previously suggested [16], this sequence logo displays a concentrated distribution where residues in ±6 range is much more discernible than those in more distal positions. Hydrophobic and small residues are favored in the proximity of Ubsites, while charged residues are under-represented. Nevertheless, it should be noted that these preferences are position-specific. Detailed discussion about the characteristic sequence patterns can be found in our previous study [16]. What we would like to address here, however, is the structural propensities of Ubsites’ context.

To address this, we first plotted the secondary structure logo of the context. This logo illustration does not show the centric distribution, and some proximal positions exhibit little secondary structure propensity (Figure 3B). Previous studies often focused on the secondary structure of Ubsites and their closest sequence neighbors only. It is therefore unexpected that even in distal positions like positions +22 ~+25, there exist discernible secondary structure propensities. Moreover, because eight-type DSSP secondary structure assignment [42] was applied here, we were able to identify more subtle details. Previous analyses suggested that coils were favored but helices were disfavored for yeast Ubsites [18]. Our results coincided with this observation, and further showed that the most favored coil type is the highly curved coil (S). Besides, distinct types of helices also exhibit different propensities. While α-helix (H) is widely depleted in the context, the 3^10^-helix (G) is somewhat favored at the proximal positions of Ubsites (Figure 3B).

The depiction of the structural propensity was enriched by introducing the structural alphabet. We plotted the 22-state structural alphabet logo in Figure 3C. Note that structural alphabet correlates but not necessarily coincides with the secondary structure assignment. For example, Ubsites prefer a highly curved coil conformation (V), which is in good agreement with their favored secondary structure type (S). However, no depletion of helix can be observed in this position from the structural alphabet logo. The situation is more obvious for positions -1, +5 and +6. While each of these positions favors specific structural alphabet state (Figure 3C), but little secondary structure propensity can be identified at the corresponding positions (Figure 3B). Generally, this logo exhibits the most discrete distribution, which plausibly results from the neighborhood-dependent nature of the structural alphabet. We speculate that this trait may be efficiently utilized to further enhance the discriminative capability of Ubsites’ context. We will test this possibility later in the next section.

**Figure 4:**
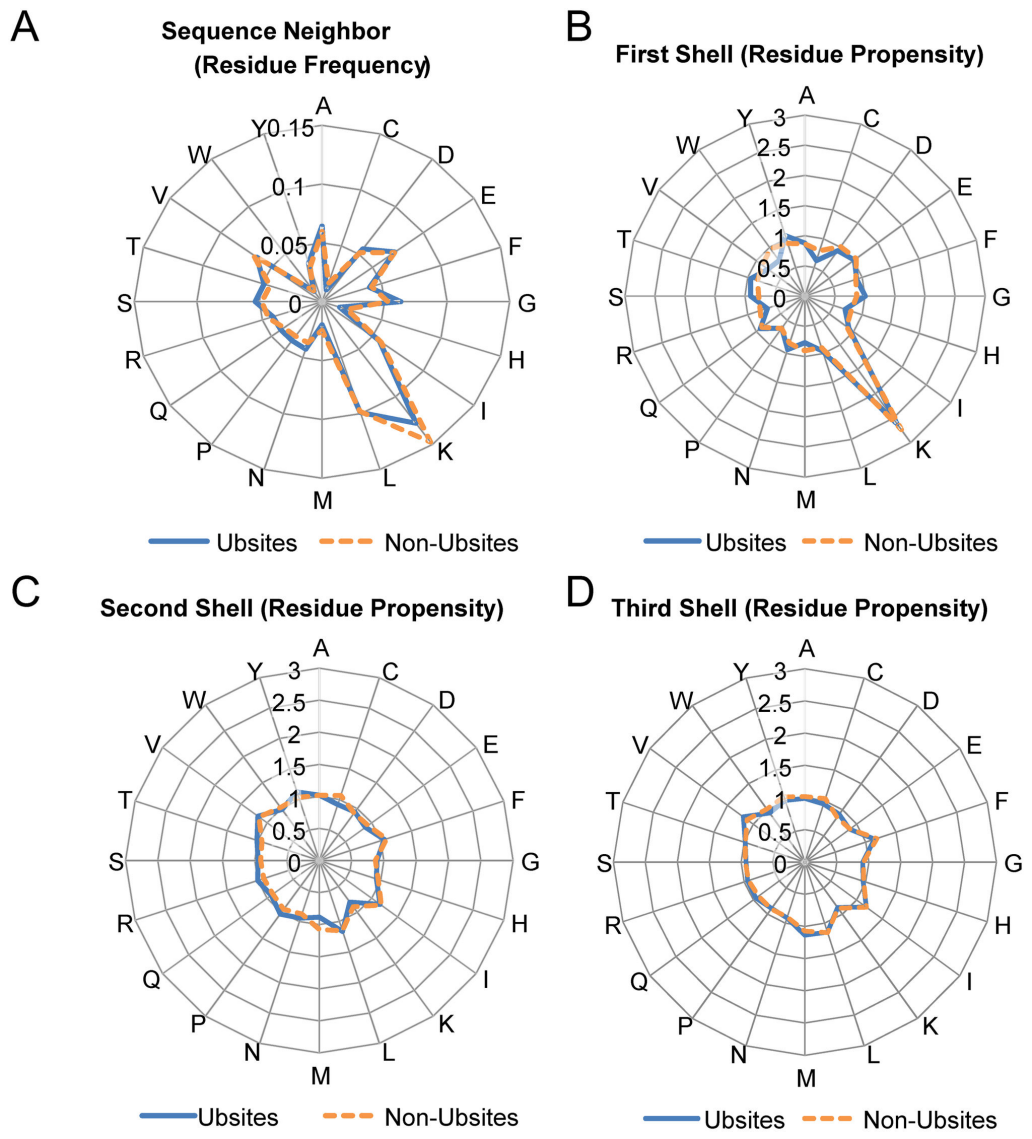
The residue usage in the proximal context and the microenvironments. (A-D) The radar diagrams which illustrate (A) the average residue frequencies in the proximal context (sequence neighbors within the ±6 residue range around the central lysine); (B) the average residue propensities in the first shell (C_β_ distance, 0Å~7.5 Å proximal to the central lysine); (C) the average residue propensities in the second shell (C_β_ distance, 7.5Å~11.5 Å); (D) the average residue propensities in the third shell (C_β_ distance, 11.5Å~15.5 Å).

In addition to the context, we defined a three-shell microenvironment for each Ubsite or Non-Ubsite. For each shell, the average amino acid propensities were calculated and plotted (Figure 4B to D). For comparison, we also plotted the average residue frequency of the proximal context (±6 residues; see Figure 4A). We observed that for the first shell, the residue propensities qualitatively agreed well with the residue frequencies of the context (Figure 4A and B). Similar results were obtained for the second shell with the exception of the enrichment of arginine (Figure 4C). The discrepancy between Ubsites and Non-Ubsites appears to be marginal for the second shell, and almost disappears for the third shell (Figure 4D). Therefore, the residue usage in the microenvironment of Ubsites appears to be distinguishable, within the scope of the first two shells.

### Sequence Pattern and Structural Propensities Are Complementary Indicators of Ubiquitination Sites

#### Structural Propensities Are Non-random Features of Ubiquitination Sites

One may note that the differences between Ubsites and Non-Ubsites in the structural propensities are not intuitively prominent. However, this by no means implies uselessness of the structural propensities. Our two computational analyses based on 10,000 artificial samples (see Text S1) indicate that a difference is unlikely to be achieved by random feature values (Figure S4A) or induced by random noise (Figure S4B), when it meets a stringent *p*-value cutoff (i.e. *p*<5.0×10^-5^). Therefore, most of the structural propensities should be considered as non-random features of Ubsites. It is also worth mentioning that our estimation of the differences in the structural propensities is conservative, since there could be other PTM sites and undiscovered Ubsites annotated as Non-Ubsites in our dataset. For example, after removing Acesites and possible undiscovered Ubsites (the Non-Ubsites whose proximal context sharing 50% or more sequence identity to that of any Ubsite), the difference between Ubsites and Non-Ubsites in CX could be further amplified (from *p*=9.9×10^-17^, *r*=-0.10 to *p*=3.8×10^-25^, *r*=-0.13). Thus, we expect higher usefulness of structural propensities, when the knowledge of PTM sites becomes more completed.

#### Structural Propensities Are Complementary to Sequence Pattern

We tested the complementary relationship between sequence pattern and structural propensities using ROC analysis. The ROC analysis is frequently used for predictor assessment. However, here it was introduced for a distinct purpose (i.e. quantifying the complementary relationship) because we do not aim at developing a new Ubsite predictor in this work. Based on the ROC analysis, several structural propensities are suggested as moderate indicators of Ubsites, and they substantially complement the information embedded in the sequence pattern.

**Figure 5:**
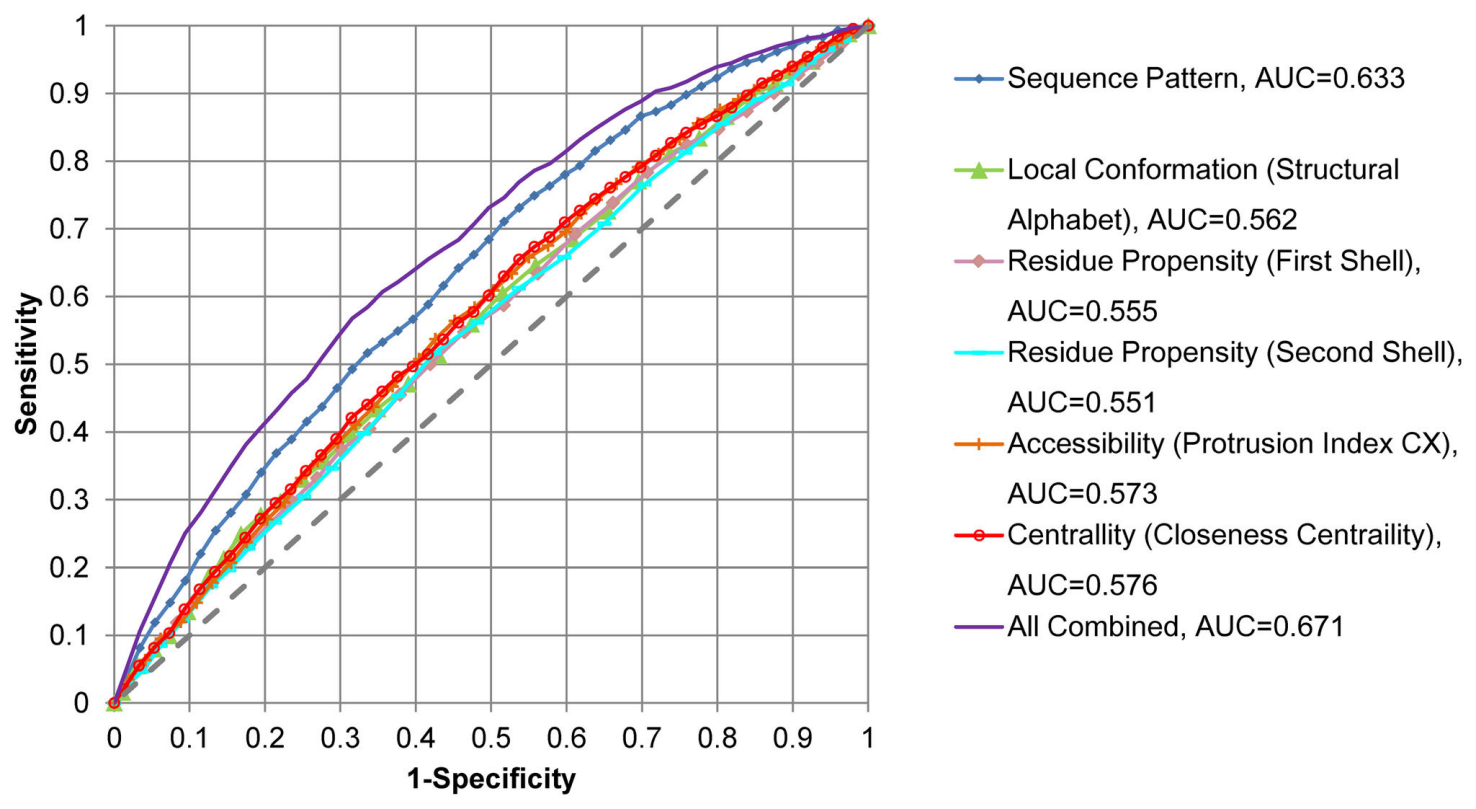
The ROC curves measuring the discriminative capability of the ubiquitination site indicators. The indicators include the sequence pattern, the structural propensities (local conformation, residue propensities in the microenvironment, accessibility and centrality) and their combination. For combination, individual indicators were combined by a weighted summing scheme (see Table S2 for the weights). The AUC values were calculated according to the structural propensities, the likelihood scores derived via five-fold cross-validation of the corresponding models or their combinations (see Text S1 for details). The larger the AUC value, the stronger the indicator.

We first assigned the likelihood score for ubiquitination according to the positional sequence pattern of the proximal context (±6 residues). This sequence pattern-derived likelihood score is the best single indicator of Ubsites in current analyses (AUC=0.633; Figure 5), in agreement with previous conjectures and results [13-16,19]. We next generated the likelihood score based on the local conformation (structural alphabet) frequencies within the same range. This local conformation-derived likelihood score is a moderate indicator of Ubsite (AUC=0.562). Similarly, the sequence propensities in the first two shells of the microenvironment could also help distinguish Ubsites, though the discriminative capability seemed to be limited according to current ROC analysis results (Figure 5). More interestingly, the accessibility and centrality indicators have achieved noticeable discriminative capability (AUC=0.573 and 0.576, respectively), in contrast to their relatively simple calculation formulae. Finally, the aforementioned six indicators, when combined together, could achieve a significant improvement of discriminative capability compared with the sequence pattern-derived likelihood score alone (AUC 0.673 *vs.* 0.633, DeLong’s test, *p*=1.9×10^-13^; Figure 5). These quantitative results highlight the complementary relationship between the sequence pattern and the structural propensities.

#### Structural Propensities Do Not Result from Sequence or Structural Redundancy

Another concern about the observed structural propensities might be raised from the de-redundancy criterion used to compile our dataset. That is, the 50% sequence identity cutoff would be too high to filter against redundant sequences and structures. Therefore, to further validate our results, we have constructed two additional datasets using more stringent de-redundancy criteria.

For the first dataset, a 30% sequence identity cutoff was applied. The numbers of resultant chains, Ubsites and Non-Ubsites of this dataset are presented in Figure S5. Intuitively, such a strict identity cutoff did not result in a dramatic shrinkage of the sample size. In fact, we found that the sample size could be largely kept across a wide range of sequence identity cutoffs (Figure S5A), implying that most sequences in our main dataset (i.e. the dataset using 50% sequence identity cutoff) are indeed non-redundant. Results based on this validation dataset indicate that our conclusions are not likely to be influenced by the alteration of the sequence identity cutoff. That is, Ubsites tend to have significantly higher accessibility and centrality, as measured by the protrusion index CX and the closeness centrality, respectively (*p*<10^-10^; Figure S6A and B). According to the ROC analysis, the local conformation and the microenvironment also exhibit marginal but detectable differences, thereby facilitating the discrimination of Ubsites from Non-Ubsites (Figure S6C). As indicated by the highest AUC of the combined indicator (Figure S6C), the ROC analysis also validates the complementary relationship between the structural propensities and the sequence pattern.

We further generated the second validation dataset by discarding redundant structures. We used the TM-align tool [55] to compare PDB chains through pair-wise structure alignments. If two PDB chains shared significant structural similarity (i.e. TM-score>0.5), only one of them would be retained. Note that this structural similarity cutoff can ensure that most of proteins in the second validation dataset do not share the same structural fold [56]. Not surprisingly, by applying this rigorous de-redundancy criterion, the sample size decreased considerably (Figure S5). Nevertheless, the significant structural propensities of Ubsites and their complementary relationship to the sequence pattern were observed again (Figure S7).

In summary, the observed structural propensities of Ubsites are unlikely to be artifacts caused by a specific de-redundancy criterion. It is argued that 50% sequence identity is an acceptable threshold to reduce the redundancy while maintaining a sizable dataset that facilitates our comprehensive analyses.

## Conclusions

The underlying mechanism of Ubsite selection has been a long-standing question. Thanks to the rapid growth of ubiquitination proteome data and protein structure information, we performed systematic analyses and demonstrated the structural propensities of Ubsites, which include accessibility, centrality and local conformation. Moreover, our analyses have revealed wide associations between Ubsites and multiple functional sites in the structures. Our quantitative analysis also clearly demonstrates that the structural propensities complement the sequence pattern to influence Ubsite specificity. Because most of current Ubsite predictors solely rely on sequence-derived information, we anticipate that such a complementary relationship may be efficiently exploited to improve the performance of dedicated Ubsite prediction tools. Further, considering some structural propensities and functional site associations observed in this study have rarely been tested for other PTM sites, we also expect that these propensities and associations will be further interrogated for other PTM sites in the future, in order to uncover the structural-level selection mechanisms of PTM sites. Last but not least, we hope that our computational pipeline can be readily applied to analyze other types of functional sites and proved useful to gain comprehensive structural insights into these functional sites.

## Supporting Information

Figure S1 **The difference between Ubsites and Non-Ubsites in accessibility and centrality using alternative parameters.** (A) Average protrusion index CX; (B) Maximum depth index DPX; (C) Average depth index DPX; (D) Closeness centrality in the residue contact networks (RCNs) generated using another definition of residue contact (i.e. two residues are considered as a contacting pair if the distance between any two atoms from each residue is smaller than 4.0 Å). Note that the ranges of whiskers (dashed lines) in all boxplots were doubled to avoid displaying too many outliers.
(TIF)

Figure S2 **Boxplots illustrating the distance between a Ubsite/Non-Ubsite and the protein geometric center.** (A) Absolute Euclidian distance; (B) Distance corrected for the protein size using the radius of gyration, large outliers (including 3 Ubsites and 26 Non-Ubsites) were not shown for clarity.
(TIF) (TIF)

Figure S3 **The ΔASA distribution of Ubsites and Non-Ubsites for different groups of protein complexes.** Residues on the interface cores are featured in high ΔASA (i.e. >85 Å^2^). (A) The ΔASA distribution of Ubsites. (B) The corresponding distribution of Non-Ubsites.
(TIF)

Figure S4 **The distribution of Wilcoxon test *p*-value among the 10,000 trails using artificial samples.** (A) The *p*-value deduced from the comparison of artificial samples with random values. (B) The *p*-value deduced from the comparison of artificial samples with random noise added.
(TIF)

Figure S5 **Sample sizes of the datasets with different de-redundancy criteria.** This figure shows (A) the numbers of PDB chains that are retained when different sequence identity cutoffs are applied, and the sample sizes of our main dataset and two additional validation datasets, in terms of (B) the number of PDB chains, (C) the number of Ubsites and (D) the number of Non-Ubsites.
(TIF)

Figure S6 **Validation of structural propensities using a dataset with 30% sequence identity cutoff.** (A) Boxplots illustrating the difference between Ubsites and Non-Ubsites in the protrusion index CX. (B) Boxplots illustrating the difference in the closeness centrality. Note that the ranges of whiskers (dashed lines) in all boxplots were doubled to avoid displaying too many outliers. (C) The ROC curves measuring the discriminative capability of the individual Ubsite indicators and their combination. The AUC values were calculated according to the structural propensities, the likelihood scores derived via five-fold cross-validation of the corresponding models or their combinations (see Text S1 for details). For combination, individual indicators were combined by a weighted summing scheme (see Table S2 for the weights). The combined indicator is significantly more powerful than the sequence pattern indicator alone (DeLong’s test, *p*= 1.2×10^-16^).
(TIF) (TIF)

Figure S7 **Validation of structural propensities using a dataset without structural redundancy.** (A) Boxplots illustrating the difference between Ubsites and Non-Ubsites in the protrusion index CX. (B) Boxplots illustrating the difference in the closeness centrality. Note that the ranges of whiskers (dashed lines) in all boxplots were doubled to avoid displaying too many outliers. (C) The ROC curves measuring the discriminative capability of the individual Ubsite indicators and their combination. The AUC values were calculated according to the structural propensities, the likelihood scores derived via five-fold cross-validation of the corresponding models or their combinations (see Text S1 for details). For combination, individual indicators were combined by a weighted summing scheme (see Table S2 for the weights). The combined indicator is significantly more powerful than the sequence pattern indicator alone (DeLong’s test, *p*= 2.3×10^-10^).
(TIF)

Table S1 **The ubiquitination sites and non-ubiquitination sites in our dataset.**
(XLS)

Table S2 **The weights used to sum the values of individual indicators into the combined scores.**
(DOC)

Text S1 **The supplementary methods describe (1) How the distance of one lysine to the protein center is calculated and corrected; (2) The analysis of folding hotspots; (3) The details of the protein complex analysis; (4) How the statistical significance is empirically validated; (5) The details of likelihood score calculation, which is part of the ROC curve analysis.**
(DOC)
