# Supplementary figures and images for: Structural Propensities of Human Ubiquitination Sites: Accessibility, Centrality and Local Conformation
Source: PLoS One. 2013 Dec 11;8(12):e83167. doi: 10.1371/journal.pone.0083167 (PMC3859641; doi:10.1371/journal.pone.0083167)

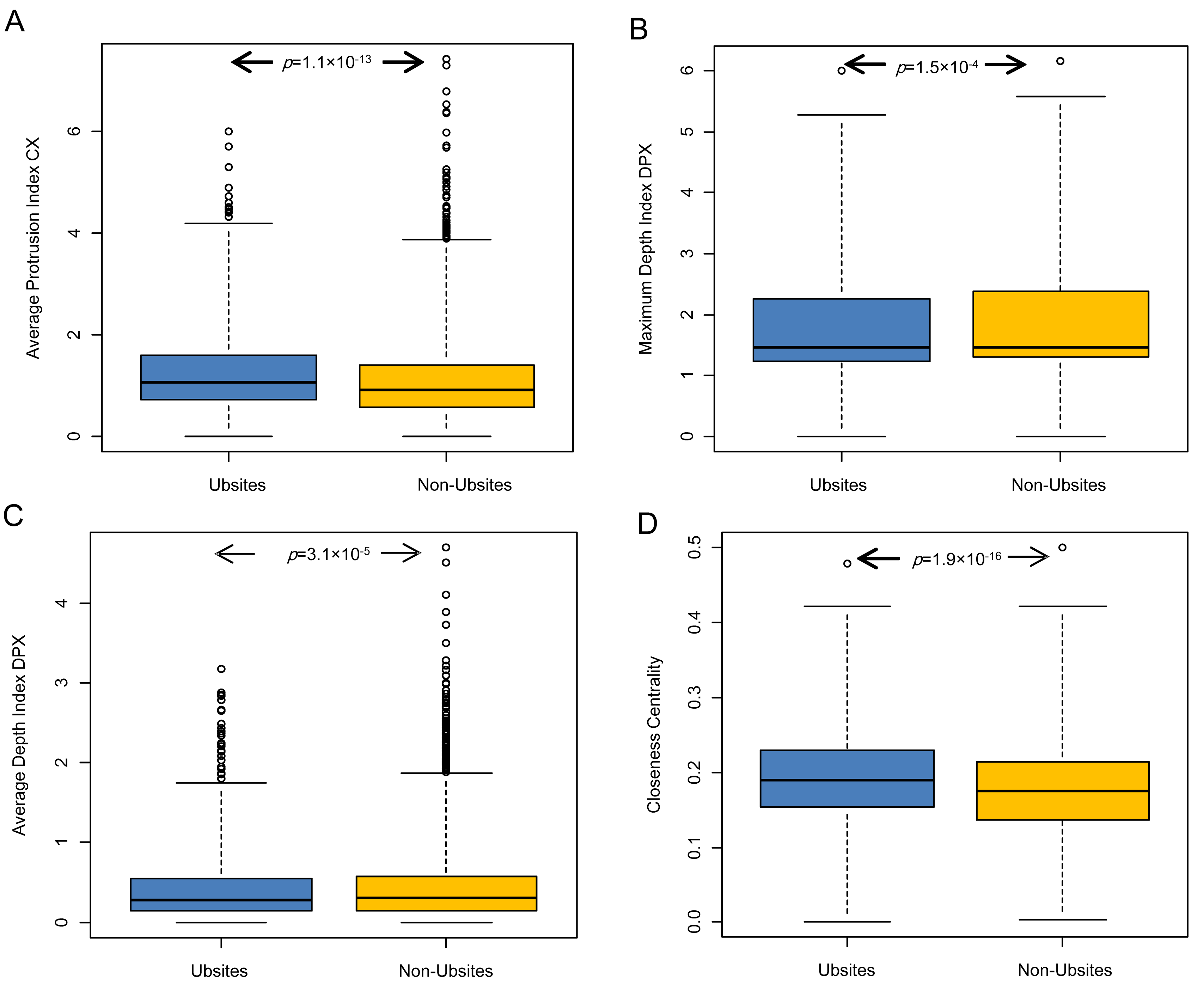

Supplement: Figure S1 — The difference between Ubsites and Non-Ubsites in accessibility and centrality using alternative parameters. (A) Average protrusion index CX; (B) Maximum depth index DPX; (C) Average depth index DPX; (D) Closeness centrality in the residue contact networks (RCNs) generated using another definition of residue contact (i.e. two residues are considered as a contacting pair if the distance between any two atoms from each residue is smaller than 4.0 Å). Note that the ranges of whiskers (dashed lines) in all boxplots were doubled to avoid displaying too many outliers. (TIF) [file pone.0083167.s001.tif]

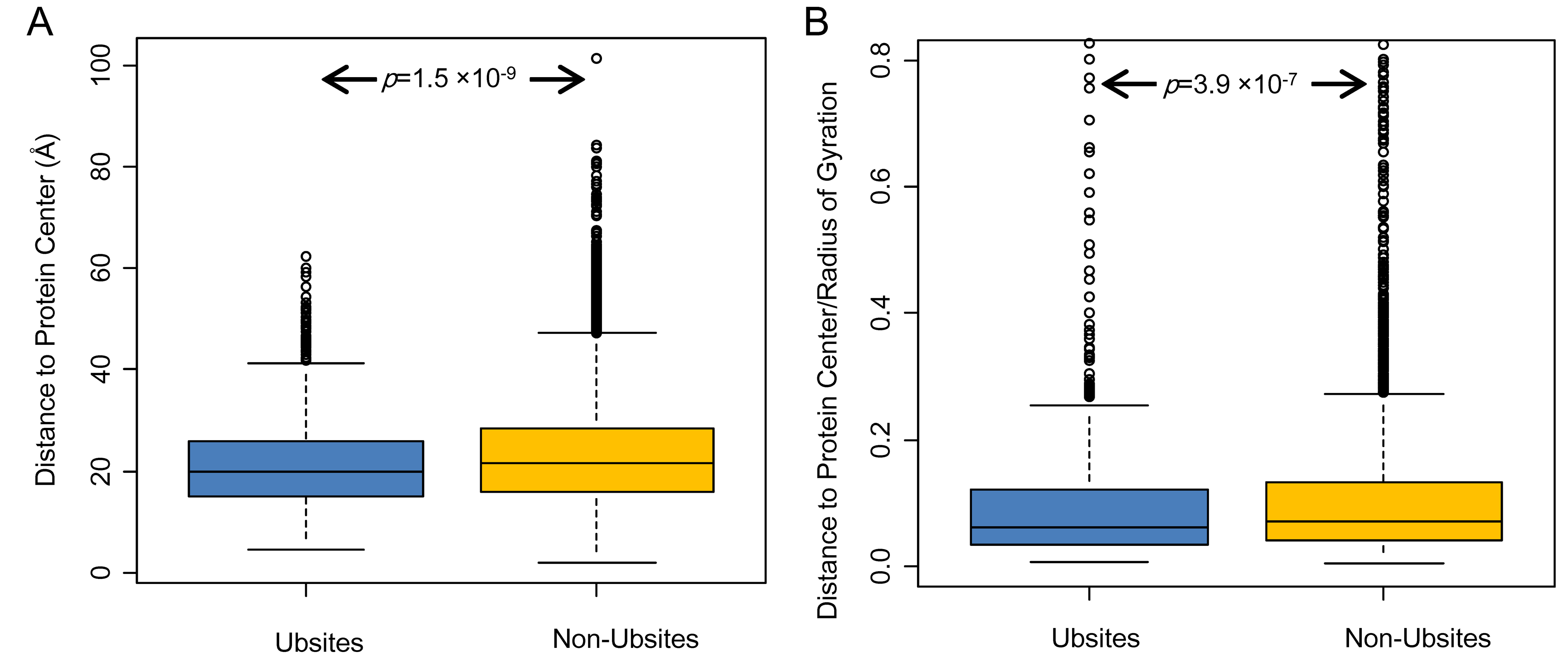

Supplement: Figure S2 — Boxplots illustrating the distance between a Ubsite/Non-Ubsite and the protein geometric center. (A) Absolute Euclidian distance; (B) Distance corrected for the protein size using the radius of gyration, large outliers (including 3 Ubsites and 26 Non-Ubsites) were not shown for clarity. (TIF) [file pone.0083167.s002.tif]

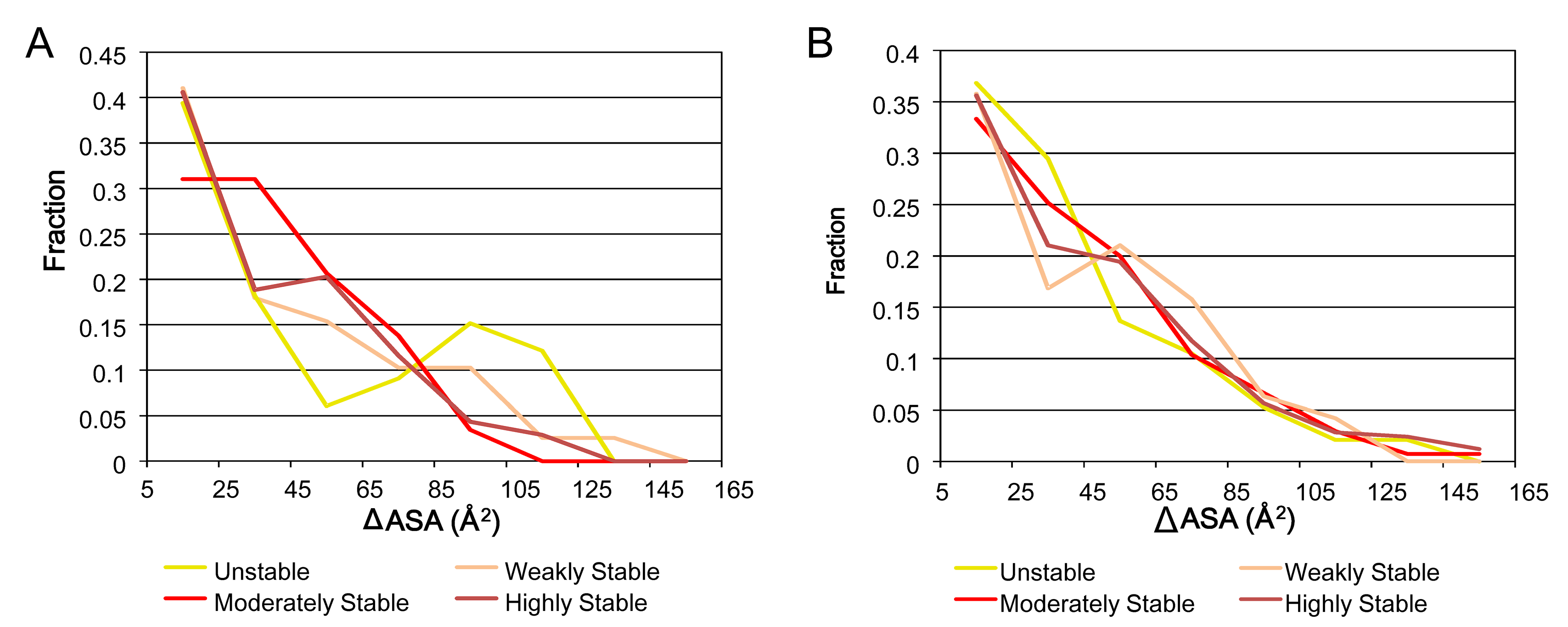

Supplement: Figure S3 — The ΔASA distribution of Ubsites and Non-Ubsites for different groups of protein complexes. Residues on the interface cores are featured in high ΔASA (i.e. >85 Å2). (A) The ΔASA distribution of Ubsites. (B) The corresponding distribution of Non-Ubsites. (TIF) [file pone.0083167.s003.tif]

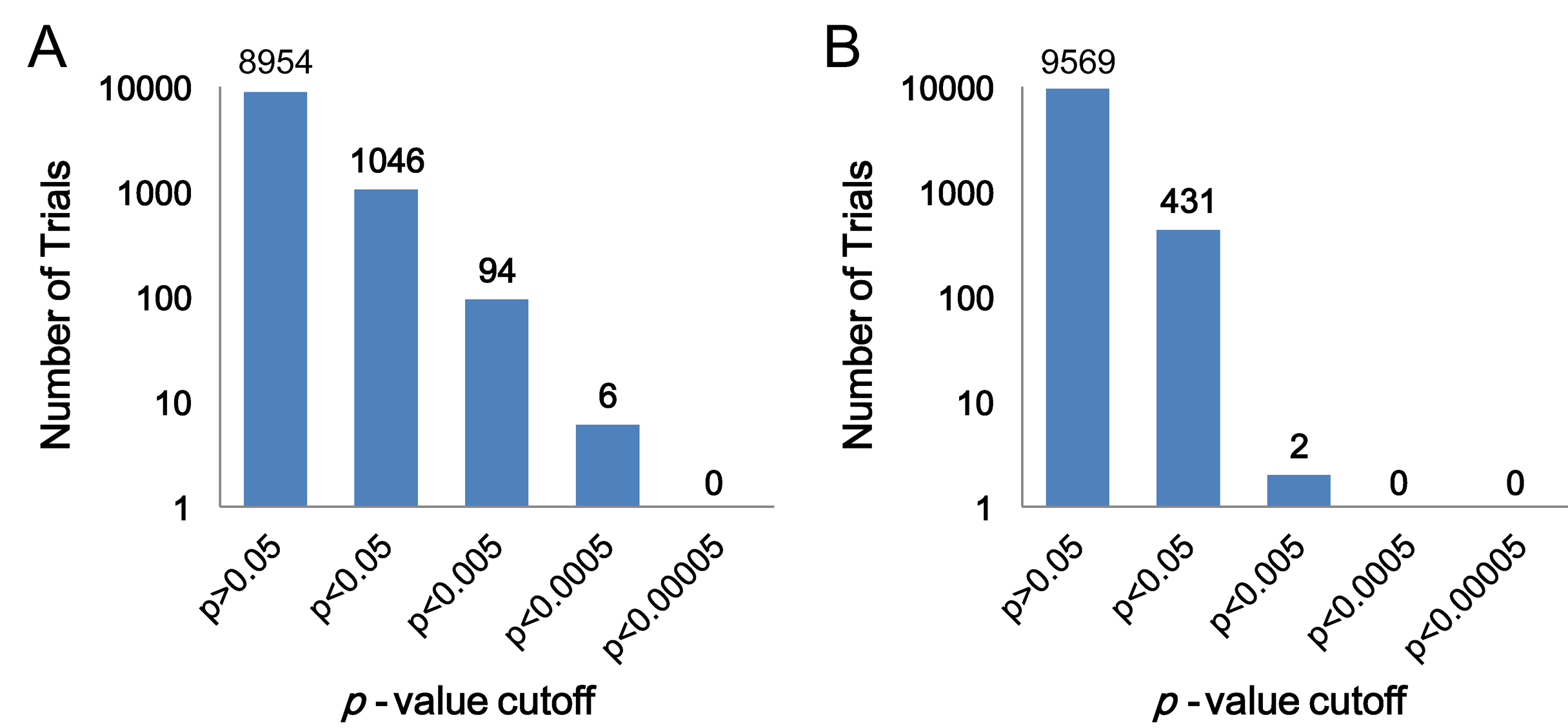

Supplement: Figure S4 — The distribution of Wilcoxon test p-value among the 10,000 trails using artificial samples. (A) The p-value deduced from the comparison of artificial samples with random values. (B) The p-value deduced from the comparison of artificial samples with random noise added. (TIF) [file pone.0083167.s004.tif]

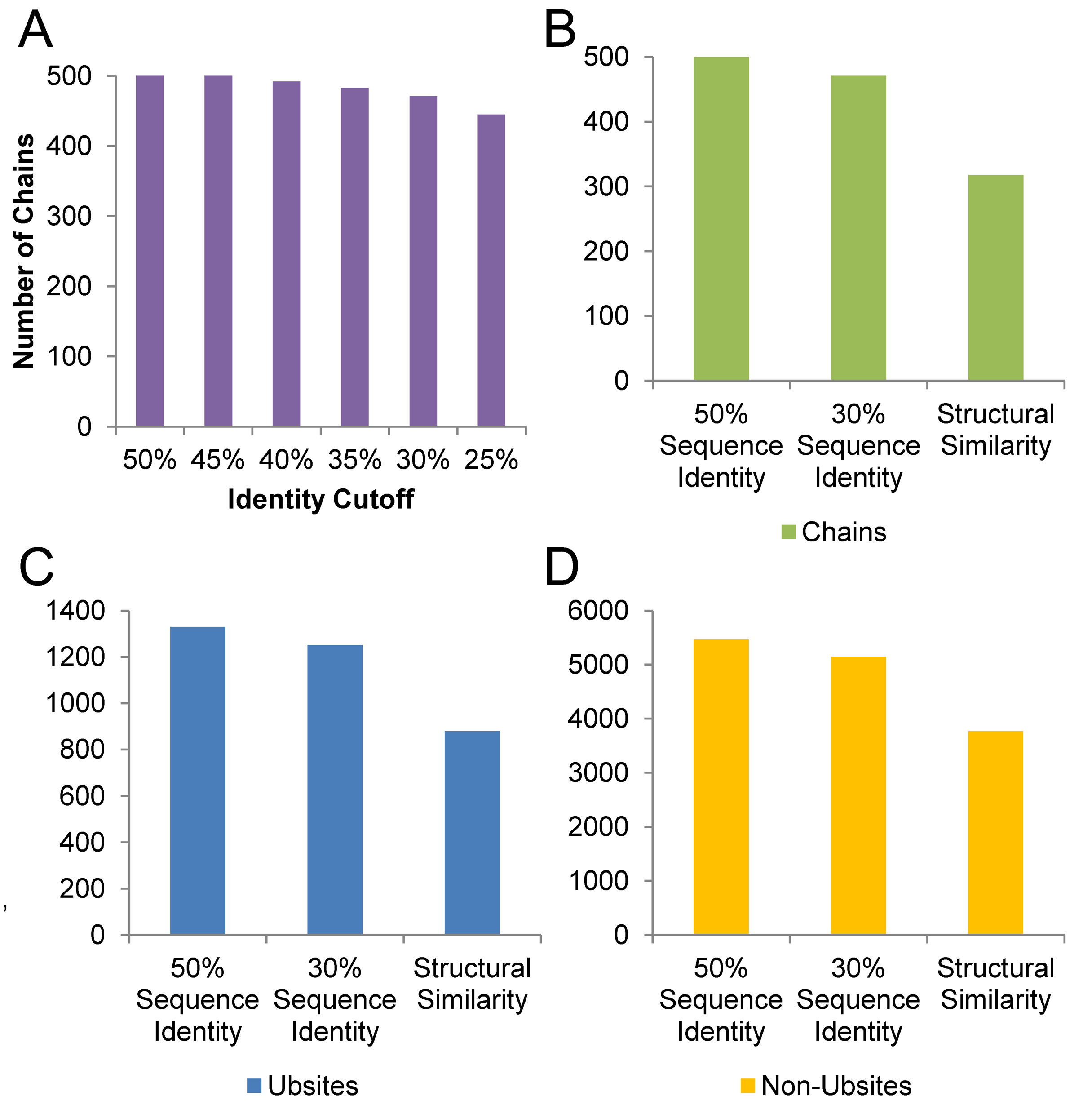

Supplement: Figure S5 — Sample sizes of the datasets with different de-redundancy criteria. This figure shows (A) the numbers of PDB chains that are retained when different sequence identity cutoffs are applied, and the sample sizes of our main dataset and two additional validation datasets, in terms of (B) the number of PDB chains, (C) the number of Ubsites and (D) the number of Non-Ubsites. (TIF) [file pone.0083167.s005.tif]

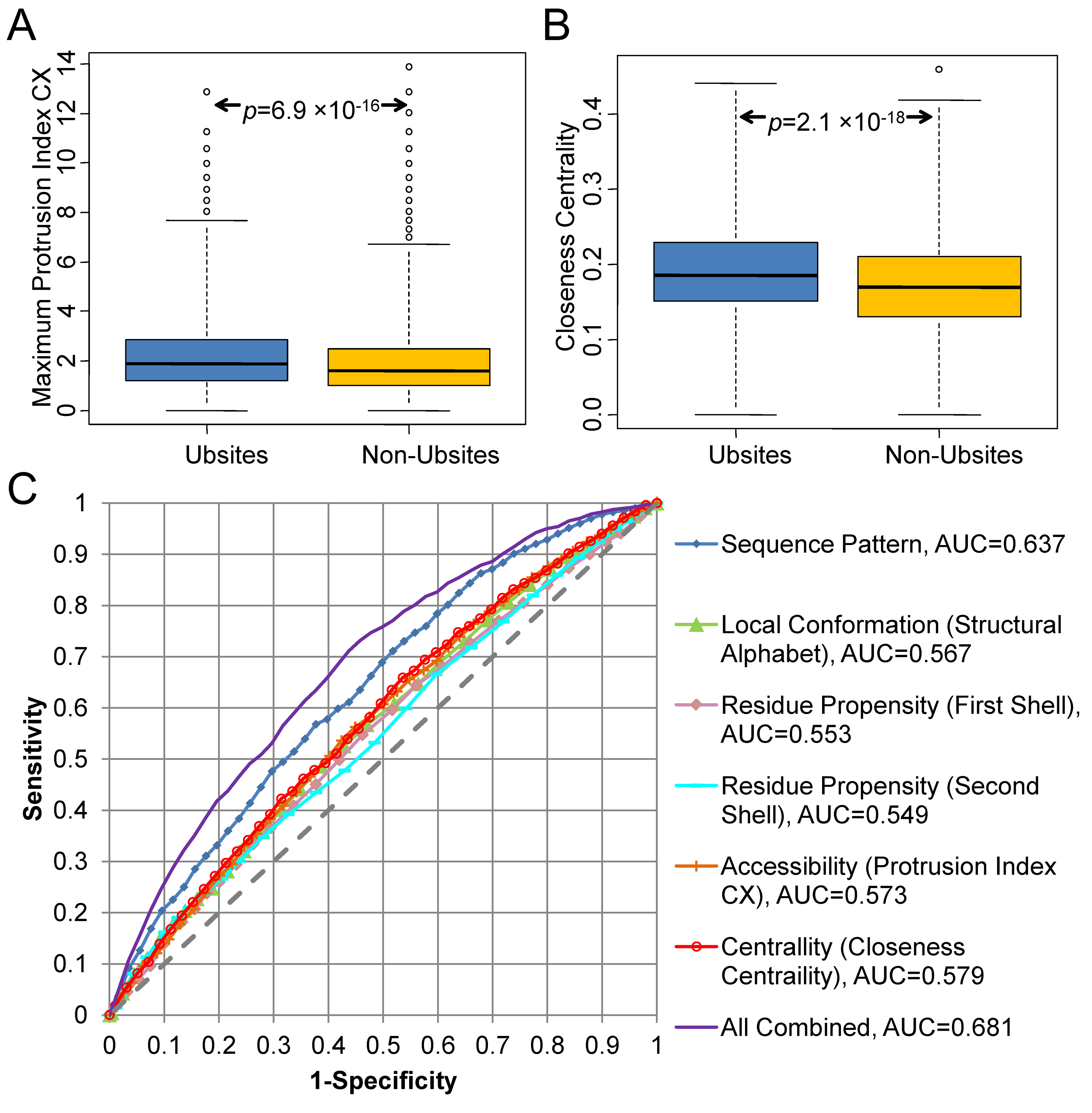

Supplement: Figure S6 — Validation of structural propensities using a dataset with 30% sequence identity cutoff. (A) Boxplots illustrating the difference between Ubsites and Non-Ubsites in the protrusion index CX. (B) Boxplots illustrating the difference in the closeness centrality. Note that the ranges of whiskers (dashed lines) in all boxplots were doubled to avoid displaying too many outliers. (C) The ROC curves measuring the discriminative capability of the individual Ubsite indicators and their combination. The AUC values were calculated according to the structural propensities, the likelihood scores derived via five-fold cross-validation of the corresponding models or their combinations (see Text S1 for details). For combination, individual indicators were combined by a weighted summing scheme (see Table S2 for the weights). The combined indicator is significantly more powerful than the sequence pattern indicator alone (DeLong’s test, p= 1.2×10^-16^). (TIF) [file pone.0083167.s006.tif]

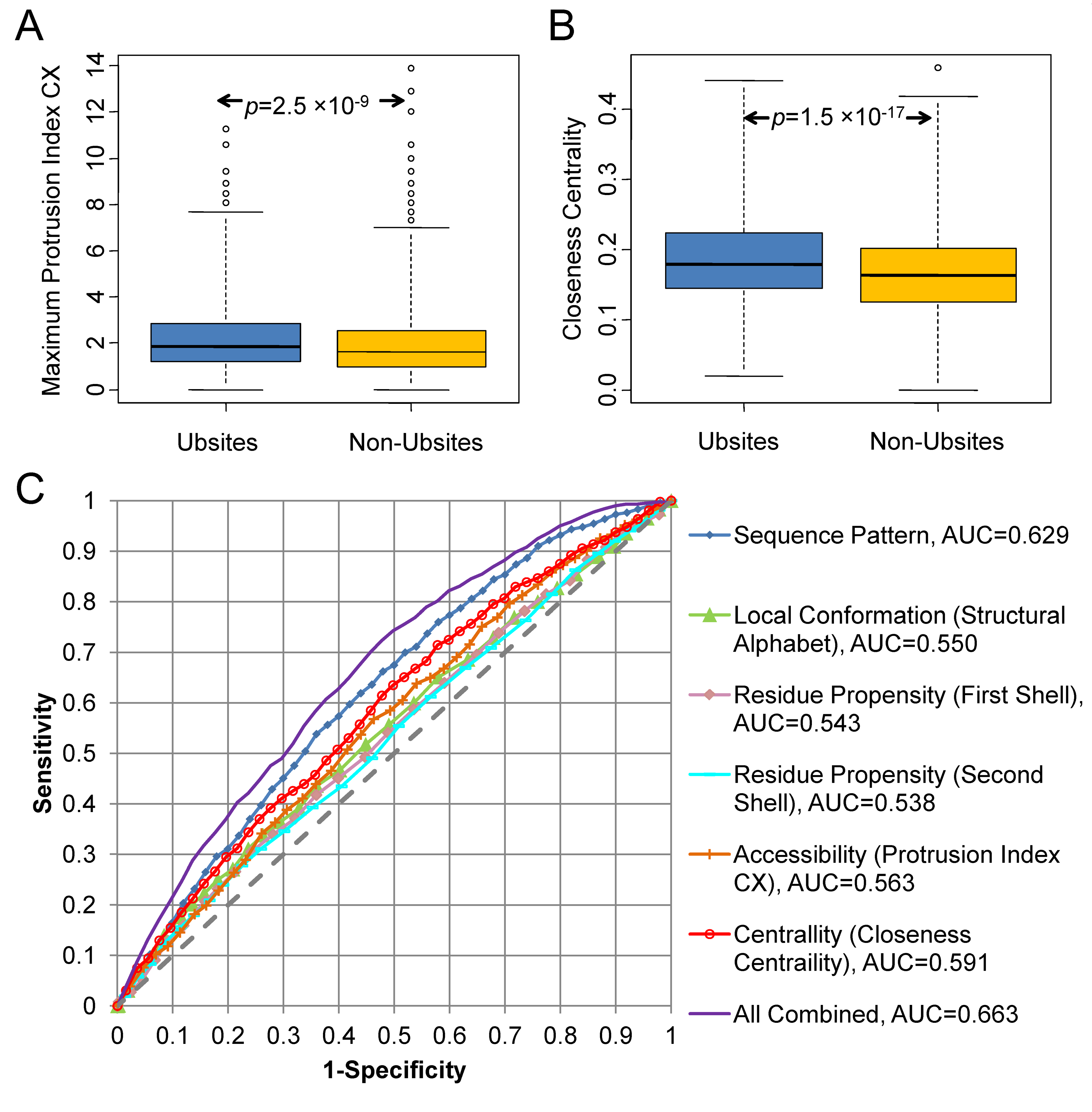

Supplement: Figure S7 — Validation of structural propensities using a dataset without structural redundancy. (A) Boxplots illustrating the difference between Ubsites and Non-Ubsites in the protrusion index CX. (B) Boxplots illustrating the difference in the closeness centrality. Note that the ranges of whiskers (dashed lines) in all boxplots were doubled to avoid displaying too many outliers. (C) The ROC curves measuring the discriminative capability of the individual Ubsite indicators and their combination. The AUC values were calculated according to the structural propensities, the likelihood scores derived via five-fold cross-validation of the corresponding models or their combinations (see Text S1 for details). For combination, individual indicators were combined by a weighted summing scheme (see Table S2 for the weights). The combined indicator is significantly more powerful than the sequence pattern indicator alone (DeLong’s test, p= 2.3×10^-10^). (TIF) [file pone.0083167.s007.tif]
