# Supplementary material for: Structural Propensities of Human Ubiquitination Sites: Accessibility, Centrality and Local Conformation
Source: PLoS One. 2013 Dec 11;8(12):e83167. doi: 10.1371/journal.pone.0083167 (PMC3859641; doi:10.1371/journal.pone.0083167)
Supplement: Table S2 — The weights used to sum the values of individual indicators into the combined scores. (DOC) [file pone.0083167.s009.doc]

**Table S2.** The weights used to sum the values of individual indicators into the combined scores.

| Individual Indicator | Weight (main dataset, 50% identity cutoff) | Weight (validation dataset 1, 30% identity cutoff) | Weight (validation dataset 2, similar structures removed) |
| --- | --- | --- | --- |
| Sequence Pattern | 0.35 | 0.30 | 0.30 |
| Local Conformation (Structural Alphabet) | 0.15 | 0.15 | 0.10 |
| Residue Propensities (First Shell) | 0.05 | 0.05 | 0.05 |
| Residue Propensities (Second Shell) | 0.05 | 0.05 | 0.05 |
| Accessibility (Protrusion Index CX) | 0.15 | 0.15 | 0.15 |
| Centrality (Closeness Centrality) | 0.25 | 0.30 | 0.35 |
