# Supplementary material for: Structural Propensities of Human Ubiquitination Sites: Accessibility, Centrality and Local Conformation
Source: PLoS One. 2013 Dec 11;8(12):e83167. doi: 10.1371/journal.pone.0083167 (PMC3859641; doi:10.1371/journal.pone.0083167)
Supplement: Text S1 — The supplementary methods describe (1) How the distance of one lysine to the protein center is calculated and corrected; (2) The analysis of folding hotspots; (3) The details of the protein complex analysis; (4) How the statistical significance is empirically validated; (5) The details of likelihood score calculation, which is part of the ROC curve analysis. (DOC) [file pone.0083167.s010.doc]

**SUPPLEMENTARY METHODS**

**Calculation of the Distance to the Protein Geometric Center**

We first extracted the coordinates of all the C_α_ atoms in a protein structure. The coordinate of the protein geometric center can be estimated by averaging the coordinates of all C_α_ atoms. Then the Euclidian distance from C_α_ atom of the each Ubsite/Non-Ubsite to the protein center was calculated . One may note that this absolute distance may be influenced by the variation of protein size, e.g., residues on a small protein are more likely to be located near the protein center than those on a large protein. To relief this potential bias, the Euclidian distance was corrected using the radius of gyration. The radius of gyration is a measurement of protein shape . Generally speaking, large or loosely packed proteins have higher radius of gyration. The corrected distance is calculated as:

where D*_Euclidian_* is the Euclidian distance; *N* is the number of residues in the protein; while *Cor_i_* and *Cor_center_* represent the coordinate vector of *i*-th C_α_ atom and that of the protein geometric center, respectively. The denominator shows a concise formula of the radius of gyration. More specifically, because only C_α_ atoms were considered during the calculation, the atom weight-related terms in the full formula were canceled accordingly.

**Computational Alanine Scan Using the FoldX Software**

To begin with, the structures were refined by the *RepairPDB* command. Then the energy contribution of each lysine residue was quantified as the protein energy change (ΔΔG) when it was mutated to alanine residue. The FoldX software provides two commands (*alascan* and *PositionScan*) for the faster and more accurate estimation of ΔΔG, respectively. We found that the results of two commands correlated well (R^2^>0.6), but the *alascan* command tends to slightly overestimate the energy contribution. Therefore, we used the *PositionScan* command to calculate the ΔΔG for each lysine residue. We used the default parameters with the only exception that we allowed the software to optimize the choice of water environment because solvent is absent in some structures.

**Classification of Protein Complexes and Interface Propensity Calculation**

We grouped the protein complexes into four groups (unstable, weakly stable, moderately stable and highly stable) according to their dissociation energy (stability) inquired from the PISA server as: <0 kcal/mol, unstable; 0~10 kcal/mol, weakly stable; 10~20 kcal/mol, moderately stable; >20 kcal/mol, highly stable. The propensity of Ubsites on the interface of each complex is calculated using the following formula:

where *C_i_ (Ub)* and *C_p_ (Ub)* are the counts of Ubsites on the interface and in the whole protein complex, respectively. *C_i_ (NonUb)* and *C_p_ (NonUb)* are the counts of their counterparts for Non-Ubsites, respectively. To avoid a zero denominator, the smoothing factor *ε* is included. We set *ε* to a very small value of 0.001.

**Empirical Evaluation of the Significance of Structural Propensities Using Artificial Samples**

As shown in the main text, Ubsites and Non-Ubsites show significant differences in several structural propensities, most of which have been supported by a significant *p*-value in the Wilcoxon test. However, since the sample size is large (1,330 Ubsites and 5,465 Non-Ubsites), a small *p*-value might be achieved or induced by random noise. Therefore, two computational experiments were performed in order to obtain a rigorous *p*-value cutoff to rule out such nominally significant *p*-values. In the first experiment, we assigned random values to two sets of artificial samples (i.e. 1,330 artificial Ubsites and 5,465 artificial Non-Ubsites) and derived *p*-value by comparing them using Wilcoxon test. We repeated such a trial 10,000 times, and we observed that no trial can obtain a *p*-value less than 5.0×10^-5^ (Figure S4A). Therefore, a stringent *p*-value cutoff (i.e. *p*<5.0×10^-5^) in the real comparison between Ubsites and Non-Ubsites can rule out the difference caused by random feature values.

In the second experiment, we started from two artificial sample sets with identical normal distribution ***N*** (0,1). Then random noise ranging from -1 to 1 was added to the values of the artificial Ubsites. The difference caused by this random noise is of nominal significance and should be ruled out. We repeated this trial 10,000 times and plotted the Wilcoxon test *p*-values in Figure S4B. Again, no trial can yield a *p*-value less than 5.0×10^-5^. Therefore, a stringent *p*-value cutoff (i.e. *p*<5.0×10^-5^) in the real comparison should be able to rule out the nominal significance induced by such random noise.

**The Likelihood Score Calculation**

We utilized the ROC analysis to quantify the Ubsite indicators’ discriminative capability and their complementary relationships. In order to perform ROC analysis, some Ubsite indicators (i.e. the sequence pattern, local conformation frequencies and residue propensities in the microenvironment), which consist of multiple-dimensional features, need to be summarized and converted into the likelihood scores. For example, the sequence pattern that includes 12 features describing the residue usage at each of the ±6 positions in the context. To deal with such an indicator, we deduced an appropriate mathematical model based on the specific feature values, and extracted the likelihood scores from the established model.

More specifically, a random forest model was deduced from (a) the local conformation frequencies in the context, or (b) the residue propensities in the microenvironment. Similarly, a naïve Bayes model was employed to summarize the positional patterns of protein sequence. The random forest model and the naïve Bayes model were established using the Weka software package and the e1071 package in R, respectively. The main reason why we used these two mathematical models in this study is that no further transformation and few intentional parameter optimization is required by them. We set the tree number of random forest uniformly to 100. Intensive parameter optimizations could be helpful to further improve the models, but is beyond the scope of this report, as our purpose is *not* to establish a new Ubsite prediction tool. To achieve a robust estimation of the likelihood scores, it should also be emphasized that five-fold cross-validations were applied for all of the models.

**References for Supplementary Methods**

1. Han L, Zhang YJ, Song J, Liu MS, Zhang Z (2012) Identification of catalytic residues using a novel feature that integrates the microenvironment and geometrical location properties of residues. PLoS One 7: e41370.

2. Ivankov DN, Bogatyreva NS, Lobanov MY, Galzitskaya OV (2009) Coupling between properties of the protein shape and the rate of protein folding. PLoS One 4: e6476.

3. Schymkowitz JW, Rousseau F, Martins IC, Ferkinghoff-Borg J, Stricher F, et al. (2005) Prediction of water and metal binding sites and their affinities by using the Fold-X force field. Proc Natl Acad Sci U S A 102: 10147-10152.

4. Krissinel E, Henrick K (2007) Inference of macromolecular assemblies from crystalline state. J Mol Biol 372: 774-797.

5. Hall M, Frank E, Holmes G, Pfahringer B, Reutemann P, et al. (2009) The WEKA data mining software: an update. SIGKDD Explor Newsl 11: 10-18.
